# A nationwide population-based study on the incidence and prognosis of HER2-positive breast cancer eligible for adjuvant pertuzumab in the modern era of neoadjuvant therapy

**DOI:** 10.1016/j.breast.2026.104834

**Published:** 2026-06-08

**Authors:** Antonis Valachis, Xingrong Liu, Balazs Acs, Johan Hartman, Theodoros Foukakis, Alexios Matikas

**Affiliations:** a Department of Oncology, Faculty of Medicine and Health, Örebro University, Örebro, Sweden; b Department of Oncology/Pathology, Karolinska Institutet, Stockholm, Sweden; c Department of Clinical Pathology and Cancer Diagnostics, Karolinska University Hospital, Stockholm, Sweden; d Breast Center, Karolinska Comprehensive Cancer Center, Stockholm, Sweden

**Keywords:** Adjuvant, APHINITY, Breast cancer, Pertuzumab, Prognosis

## Abstract

**Background:**

Adjuvant pertuzumab improves the survival of patients with HER2-positive and node positive breast cancer. We aimed to evaluate the incidence and prognosis of patients eligible for adjuvant pertuzumab, both following primary resection and neoadjuvant therapy, during the modern era of preoperative treatment.

**Methods:**

This was a retrospective analysis of a prospectively collected, nationwide, population-based cohort that comprises all patients treated for breast cancer in Sweden between 2007 and 2023. Patients with non-metastatic, HER2-positive and node positive breast cancer were included in an adjuvant cohort comprising patients who underwent primary surgery, and a neoadjuvant cohort consisting of patients diagnosed with cN + disease who received neoadjuvant therapy and achieved pathologic complete response (pCR). Median follow-up was 8.2 years (interquartile range, 4.4 – 12.0).

**Results:**

Of 135080 patients diagnosed with breast cancer during the study period, 3495 were included into the adjuvant cohort. The proportion of patients eligible for adjuvant pertuzumab declined over the years. Ten-year overall survival in the adjuvant cohort was 71.6% (95% Confidence Interval [CI] 69.9 – 73.3%). Tumor size and nodal stage were independently prognostic, while estrogen receptor status was associated with late recurrence (time-dependent HR_adj_ = 1.96, 95% CI 1.21-3.19). Patients in the neoadjuvant cohort had excellent outcomes.

**Conclusions:**

Although the proportion of patients with HER2-positive and node positive breast cancer who undergo primary surgery is decreasing, their poor long-term outcomes level underscore the relevance of adjuvant pertuzumab. In contrast, the recommendation for adjuvant pertuzumab to patients that achieve pCR should be revisited considering their excellent prognosis and likely marginal absolute benefit.

## Highlights

• The modern role of adjuvant pertuzumab in HER2-positive breast cancer remains unclear.
• This is a nationwide cohort of patients with breast cancer in Sweden (2007-2023).
• The proportion of patients eligible for adjuvant pertuzumab is declining.
• Outcomes are heterogeneous, opening for both escalation and de-escalation of pertuzumab.

## 1 Introduction

The introduction of trastuzumab as part of adjuvant therapy for patients with human epidermal growth factor receptor 2 (HER2) positive breast cancer revolutionized treatment strategies and transformed a group previously associated with poor prognosis into one with outcomes comparable to less aggressive disease subtypes [1,2]. To further optimize anti-HER2 treatment, dual HER2 blockade with trastuzumab and pertuzumab has been evaluated in the adjuvant setting. In the APHINITY trial, the addition of pertuzumab led to a statistically significant, albeit modest in terms of absolute benefit, improvement in survival after a median follow-up of 11 years among patients with lymph node positive disease [3].

The implementation of this treatment strategy in clinical practice has been limited as neoadjuvant approaches, including dual HER2 blockade followed by response-guided post-neoadjuvant therapy, have become the standard of care for stage II–III HER2-positive breast cancer [[4], [5], [6]]. In addition, despite guideline recommendations from the European Society for Medical Oncology (ESMO) and National Comprehensive Cancer Network (NCCN) for the use of adjuvant pertuzumab in patients with clinically node positive (cN+) disease who achieve a pathological complete response (pCR) after neoadjuvant therapy [7,8], its role in the modern era of response-guided HER2-targeted treatment remains uncertain. Evaluating the baseline mortality risk in population-based cohorts that would theoretically be candidates for adjuvant pertuzumab, compared with cohorts treated with contemporary neoadjuvant regimens, may provide valuable insights into the potential incremental benefit of adjuvant pertuzumab.

The aim of the present study was to assess the incidence of pertuzumab eligibility, the overall survival (OS) of patients with HER2-positive disease treated with adjuvant or neoadjuvant therapy that would be potentially eligible for adjuvant pertuzumab, and to explore clinicopathological factors associated with worse survival that may identify patients suitable for escalation strategies beyond adjuvant trastuzumab.

## 2 Patients and methods

### 2.1 Study design

This is a retrospective analysis of a prospectively collected, nationwide registry cohort of all patients treated for non-invasive and invasive breast cancer in Sweden [9]. The primary objective of this study is to investigate the prevalence, patient characteristics and long-term prognosis of patients potentially eligible for adjuvant pertuzumab according to international guidelines, since pertuzumab is not reimbursed for the indication in Sweden. The population-based study was approved by the ethics review committee in Stockholm (decision 2019-01908 with amendment 2024-05048-02). The ethical approval granted a waiver for informed consent in this non-interventional collection and analysis of data from registries and patient records. The ESMO-GROW checklist was used to guide the reporting of this study [10].

### 2.2 Data sources

The National Quality Registry for Breast Cancer (Nationella kvalitetsregistret för bröstcancer; NKBC) was used as the data source to identify patients with non-metastatic HER2-positive breast cancer diagnosed between 2007 and 2023. The NKBC has a coverage exceeding 99%, capturing nearly all patients diagnosed with breast cancer in Sweden, and has been validated with high concordance rates [11]. Using the unique personal identification number assigned to all persons registered in Sweden, data from the Cause of Death Registry are periodically linked to the NKBC.

### 2.3 Patient selection

We included all patients with non-metastatic, HER2-positive breast cancer and lymph node–positive disease, either at diagnosis (cN+) or after surgery (pN1–3), who underwent surgical resection. Patients with synchronous contralateral breast cancer, defined as new contralateral cancer within three months of breast cancer diagnosis, were excluded. We defined two cohorts: an adjuvant cohort comprising patients theoretically eligible for the APHINITY trial (those who underwent primary surgery and had pN1–3 disease); and a neoadjuvant cohort consisting of patients diagnosed with cN + disease who received neoadjuvant therapy and achieved a pathologic complete response. This cohort is especially relevant given that both ESMO and NCCN guidelines recommend consideration of post-neoadjuvant dual HER2 blockade in cN + disease [7,8]. Patients diagnosed with cN + disease who received neoadjuvant therapy and had invasive residual disease at surgery were used as comparators. Within the adjuvant and neoadjuvant cohorts, “optimal treatment” sub-cohorts were defined as patients who received optimal therapy, consisting of chemotherapy and trastuzumab, and were used for sensitivity analyses.

### 2.4 Data collection and outcomes

Data concerning baseline patient characteristics, tumor pathology and administered treatment were obtained from the NKBC, while survival outcomes were retrieved from the Cause of Death Registry. Pathological complete response was defined as the absence of any invasive disease in both the breast and lymph nodes following neoadjuvant therapy and surgery. Overall survival was defined as the time from breast cancer surgery to death from any cause [12], and was chosen as the endpoint of this study since it is unequivocal not subject to misclassification or missingness in the population-based cohort which precluded the evaluation of other time-to-failure endpoints, but also to facilitate indirect comparisons with the results of the APHINITY trial.

### 2.5 Statistical analysis

Baseline clinicopathological characteristics were summarized separately for the adjuvant and neoadjuvant cohorts. Categorical variables were presented as frequencies and percentages (after excluding missing), and continuous variables as medians with interquartile ranges. No formal statistical comparison was made between the three groups.

Survival functions were estimated using the Kaplan–Meier method and compared with the log-rank test. For each comparison, 3-, 5-, 8- and 10-year survival estimates with 95% confidence intervals (CI) were reported where supported by follow-up duration and event numbers. Survival of patients receiving neoadjuvant treatment versus primary surgery was additionally compared after exploratory propensity score matching for age, mode of diagnosis (screening versus symptoms) and TNM stage. To identify clinicopathological factors associated with survival, multivariable Cox proportional hazards regression was performed. Covariates included age, estrogen receptor (ER) status, grade, T status, N status, chemotherapy type, and radiotherapy. In a sensitivity analysis, diagnostic period was added to mitigate potential effects of temporal changes in treatment guidelines and diagnostic technologies. Association results were reported as adjusted hazard ratios (HR) with 95% Confidence Intervals (CI). Complete case analysis was performed when missing covariates accounted for less than 5% of observations. The proportional hazards assumption was assessed using Schoenfeld residuals. For any variable that violated this assumption, time-dependent HRs were subsequently estimated based on flexible parametric models [13]. Additional sensitivity analyses were performed for patients treated with both chemotherapy and trastuzumab at the neoadjuvant or adjuvant settings (“optimal treatment” cohorts).

All analyses were conducted using R (version 4.4.2) and statistical significance was defined as two-sided p < 0.05.

## 3 Results

### 3.1 Patient selection and baseline characteristics

**Fig. 1:**
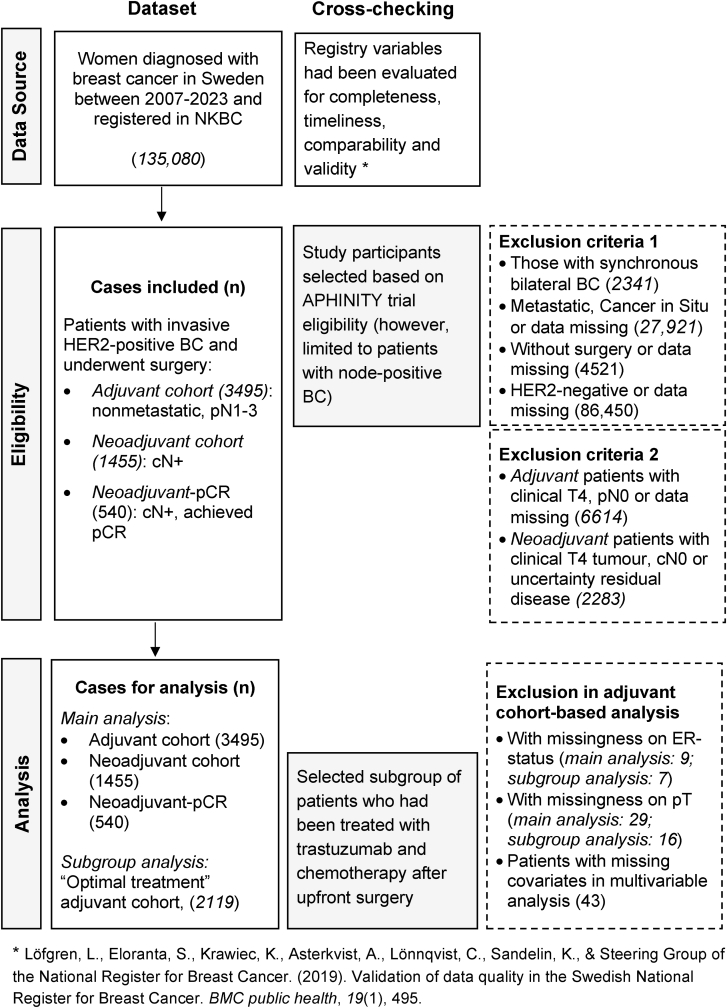
Flowchart of patient selection, reported according to the European Society for Medical Oncology Guidance for Reporting Oncology Real-World Evidence (ESMO-GROW).

Of 135080 patients diagnosed with non-invasive or invasive breast cancer of any stage in Sweden between 2007 and 2023 and registered in the NKBC, 3495 were included in the adjuvant cohort consisting of patients with non-metastatic disease (pN1–3) who received adjuvant therapy only. In addition, 1455 patients with HER2-positive and cN + breast cancer received neoadjuvant therapy during the study period, of which 540 attained pCR and 915 had residual invasive disease. A flowchart illustrating the patient selection process is presented in Fig. 1. Over time, a decrease in the number of patients included in the adjuvant cohort was observed, accompanied by a corresponding increase in the neoadjuvant cohort, regardless of nodal status Supplementary Fig. 1A–D. The proportion of patients within the adjuvant cohort who would have been eligible for pertuzumab in relation to the entire population of patients with HER2-positive breast cancer, according to APHINITY criteria, showed a slight decline over time (Supplementary Fig. 1E).

Table 1 summarizes the baseline characteristics of the adjuvant and neoadjuvant cohorts. The neoadjuvant cohort included younger patients with more anatomically advanced disease and more aggressive tumor biology. A small proportion of patients (3.6%) received adjuvant pertuzumab as part of their treatment (Supplementary Table 1). Median follow-up for the entire study cohort was 8.2 years (interquartile range, 4.4 – 12.0 years).

**Table 1:** Distribution of baseline characteristics and relevant treatments for study participants with invasive HER2-positive, node-positive breast cancer that underwent surgery, stratified by cohorts^a^. Percentages are calculated on complete data.

	Neoadjuvant-pCR, n (%)	Neoadjuvant-all n (%)	Adjuvant cohort n (%)
**No. of patients**	540	1455	3495
**Age, yrs,** median (Q1-Q3)	53 (44-62)	53 (43-64)	61 (50-71)
<40	74 (13.7)	248 (17.0)	243 (7.0)
40-49	139 (25.7)	341 (23.4)	590 (16.9)
50-64	213 (39.4)	530 (36.4)	1237 (35.4)
65-79	105 (19.4)	294 (20.2)	1030 (29.5)
≥80	9 (1.7)	42 (2.9)	395 (11.3)
**Estrogen receptor**			
Negative	329 (61.5)	606 (42.6)	1109 (31.8)
Positive	206 (38.5)	816 (57.4)	2377 (68.2)
Missing	5	33	9
**Tumor size**			
T1	102 (19.2)	221 (15.4)	1400 (40.4)
T2	291 (54.8)	825 (57.6)	1764 (50.9)
T3	138 (26.0)	386 (27.0)	302 (8.7)
Missing	9	23	29
**Nodal status**			
N1	516 (95.6)	1378 (94.7)	2383 (68.2)
N2	9 (1.7)	33 (2.3)	736 (21.1)
N3	15 (2.8)	44 (3.0)	376 (10.8)
**Tumor grade**^b^			
Grade 1-2	−	−	1179 (33.7)
Grade 3	−	−	2316 (66.3)
**Ki-67 expression, %**			
Median (Q1-Q3)	45.0 (32.0-60.0)	40.0 (30.0-58.0)	38.0 (25.0-50.5)
Missing	16	205	1084
**Type of chemotherapy**			
Taxane & anthracycline	419 (77.6)	978 (67.2)	1008 (28.8)
Taxane only	58 (10.7)	113 (7.8)	203 (5.8)
Anthracycline only	24 (4.4)	213 (14.6)	1022 (29.2)
None (or missing)	39 (7.2)	151 (10.4)	1262 (36.1)
**Trastuzumab** (administered)	465 (86.1)	1083 (74.4)	2168 (62.0)
**Pertuzumab** (administered)	418 (77.4)	817 (56.2)	80 (2.3)
**Radiotherapy target**			
Breast & Axillary	416 (77.0)	1142 (78.5)	1904 (54.5)
Breast only	48 (8.9)	119 (8.2)	489 (14.0)
None (or missing)	76 (14.1)	194 (13.3)	1102 (31.5)
**Endocrine therapy**			
Administered	185 (38.3)	774 (59.1)	1970 (67.7)
Missing	57	146	586

*Abbreviations*: HER2, human epidermal growth factor receptor 2; Q1, quartile 1; Q3, quartile 3; T, tumor; N, node.

a Baseline characteristics and treatments were determined based on relevant settings, using clinical assessments, surgical information, or details of therapies administered before or after surgery.

b Preoperative grade data was not available (indicated by the symbol “-”).

### 3.2 Prognosis of the adjuvant cohort

**Fig. 2:**
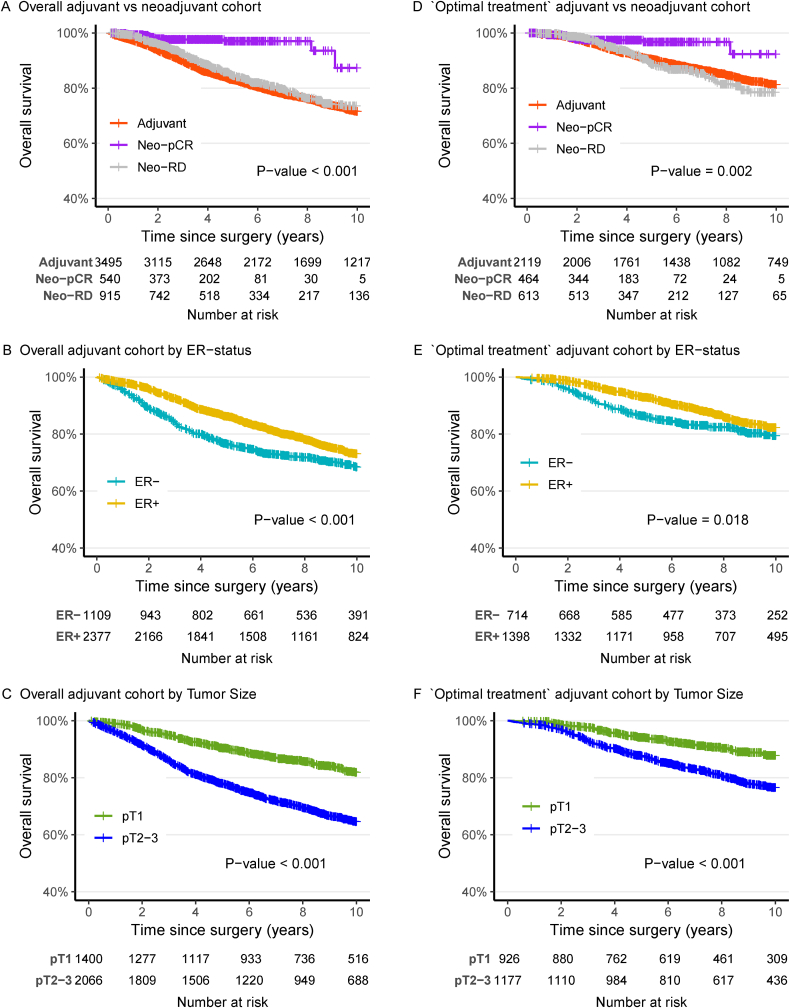
Kaplan-Meier curves for overall survival for the comparisons of the overall adjuvant versus neoadjuvant cohorts (A); estrogen receptor (ER) positive versus ER-negative disease within the adjuvant cohort (B); adjuvant versus neoadjuvant cohorts when treated with “optimal treatment” (C); ER-positive versus ER-negative disease with the adjuvant cohort treated with “optimal treatment” (D). P values refer to log-rank test.

Patients in the adjuvant cohort had 10-year survival rates of 71.6% (95% CI 69.9 – 73.3%). Within both the overall and “optimal treatment” adjuvant cohorts, ER-negative status and larger tumor size (T2–T3) were associated with inferior survival (Fig. 2). For instance, patients with small (pT1) node positive tumors that had received chemotherapy and trastuzumab had 10-year OS rates of 87.9% (95% CI 85.3 – 90.4%), compared to 76.6% for those with pT2/T3 tumors (95% CI 73.9 – 79.4%, log-rank p < 0.001; Table 2). Survival differed significantly according to age, with patients ≥70 years exhibiting worse OS than patients <70 years (log-rank p < 0.001), owing at least partly to lesser usage of adjuvant trastuzumab (43% versus 70%). However, the difference in outcomes remained even among patients receiving optimal treatment (Supplementary Fig. 2).

**Table 2:** Overall survival rates and 95% CIs presented across subgroups of interest, based on the entire study population, the adjuvant cohort only and its subset of participants treated with both chemotherapy and trastuzumab.

Overall adjuvant cohort							
Setting	Group	No.	3-year OS, %	5-year OS, %	8-year OS, %	10-year OS, %	Log-rank test
Adjuvant & neoadjuvant	Adjuvant cohort	3495	89.8	83.1	76.1	71.6	p < 0.0001
			(88.7-90.8)	(81.8-84.4)	(74.5-77.6)	(69.9-73.3)	
	Neoadjuvant-all cohort	1455	94.2	88.2	81.0	77.5	
			(92.9-95.6)	(86.1-90.3)	(77.9-84.2)	(73.9-81.4)	
	Neoadjuvant-pCR cohort	540	97.6 (96.2-99.1)	97.0 (95.1-98.9)	97.0 (95.1-98.9)	87.3 (74.8-100)	−
Adjuvant only	ER-negative	1109	84.0	76.6	71.8	68.4	p < 0.0001
			(81.8-86.2)	(74.1-79.2)	(69.0-74.6)	(65.5-71.5)	
	ER-positive	2377	92.5	86.2	78.1	73.0	
			(91.4-93.6)	(84.7-87.6)	(76.3-79.9)	(71.0-75.1)	
Adjuvant only	pT1	1400	95.0	90.4	85.8	81.9	p < 0.0001
			(93.8-96.2)	(88.8-92.1)	(83.8-87.8)	(79.6-84.3)	
	pT2-3	2066	86.2	78.0	69.5	64.6	
			(84.7-87.7)	(76.2-79.9)	(67.4-71.7)	(62.3-67.0)	

“Optimal treatment” adjuvant cohort							
Cohort	Comparison group	No.	3-year OS, %	5-year OS, %	8-year OS, %	10-year OS, %	Log-rank test
Adjuvant & neoadjuvant	Adjuvant	2119	95.0	90.6	84.7	81.3	p = 0.326
			(94.1-96.0)	(89.3-91.9)	(83.1-86.5)	(79.4-83.3)	
	Neoadjuvant-all	1077	96.5	91.3	85.4	82.1	
			(95.3-97.8)	(89.1-93.6)	(81.8-89.1)	(77.8-86.8)	
	Neoadjuvant-pCR	464	97.4 (95.8-99.0)	96.7 (94.7-98.8)	96.7 (94.7-98.8)	92.3 (84.1-100)	−
Adjuvant only	ER-negative	714	91.5	86.2	82.4	79.4	p = 0.018
			(89.5-93.6)	(83.6-88.8)	(79.5-85.4)	(76.2-82.8)	
	ER-positive	1398	96.8	92.9	85.9	82.3	
			(95.9-97.8)	(91.5-94.3)	(83.9-88.0)	(79.9-84.7)	
Adjuvant only	pT1	926	97.6 (96.7-98.6)	94.2 (92.6-95.8)	90.3 (88.2-92.5)	87.9 (85.3-90.4)	p < 0.0001
	pT2-3	1177	92.9	87.8	80.6	76.6	
			(91.5-94.4)	(85.9-89.7)	(78.2-83.1)	(73.9-79.4)	

*Abbreviations:* CI, confidence interval; OS, overall survival; ER, Estrogen Receptor; pT, pathological primary tumor.

**Fig. 3:**
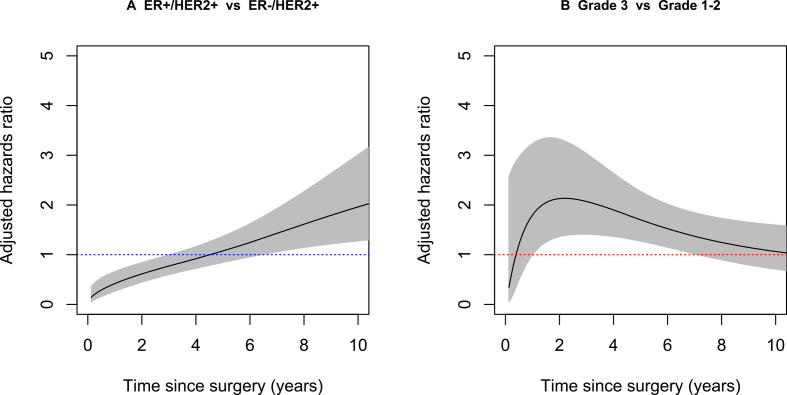
Smoothed hazards plots for death according to estrogen receptor status (A) and grade (B) in the overall adjuvant cohort. Abbreviations: ER: estrogen receptor; HER2: human epidermal growth factor receptor 2.

As ER status and tumor grade violated the proportional hazards assumption, time-dependent analyses were performed. These confirmed tumor size and nodal status as consistent predictors of survival, while also indicating a potential increase in mortality risk for patients with ER-positive disease at approximately 8 and 10 years of follow-up, and an elevated mortality risk for grade 3 tumors during the first 5 years after diagnosis (Table 3; Fig. 3). Sensitivity analysis of patients that received adjuvant chemotherapy and trastuzumab confirmed these findings Supplementary Tables 2 and 3.

**Table 3:** Univariate and multivariable analyses on association of baseline characteristics with overall survival among the total adjuvant cohort (n = 3495).

	Hazard ratio (95% CI)		
	Univariate analysis, PH models	Multivariable analysis, PH model 1	Multivariable analysis, non-PH model 1
**Age, yrs**	1.068 (1.062-1.074)	1.053 (1.046-1.060)	1.053 (1.047-1.060)
**Estrogen receptor**			
Negative	1.0 (reference)	1.0 (reference)	1.0 (reference)
Positive	0.79 (0.69-0.90)	0.93 (0.81-1.07)	Time-dependent HRs *at 2 years since surgery:* 0.64 (0.52-0.77) *at 5 years since surgery:* 1.05 (0.90-1.24) *at 8 years since surgery:* 1.61 (1.27-2.04) *at 10 years since surgery* 1.98 (1.47-2.68)
**Tumor grade**			
Grade 1-2	1.0 (reference)	1.0 (reference)	1.0 (reference)
Grade 3	1.42 (1.23-1.64)	1.29 (1.11-1.50)	Time-dependent HRs *at 2 years since surgery:* 1.77 (1.41-2.22) *at 5 years since surgery:* 1.32 (1.12-1.55) *at 8 years since surgery:* 1.04 (0.86-1.27) *at 10 years since surgery* 0.93 (0.74-1.17)
**Tumor size**			
T1	1.0 (reference)	1.0 (reference)	1.0 (reference)
T2	1.89 (1.62-2.20)	1.38 (1.18-1.62)	1.40 (1.19-1.63)
T3	3.49 (2.48-4.30)	2.34 (1.88-2.92)	2.27 (1.82-2.83)
**Nodal status**			
N1	1.0 (reference)	1.0 (reference)	1.0 (reference)
N2	1.86 (1.60-2.16)	1.55 (1.33-1.82)	1.59 (1.35-1.86)
N3	3.10 (2.62-3.67)	2.44 (2.04-2.93)	2.47 (2.06-2.97)
**Chemotherapy type**			
Anthracycline and Taxane	1.0 (reference)	1.0 (reference)	1.0 (reference)
Anthracycline only	1.80 (1.39-2.33)	1.57 (1.19-2.08)	1.55 (1.17-2.06)
Taxane only	3.23 (2.25-4.66)	1.52 (1.04-2.22)	1.48 (1.02-2.16)
None	5.07 (4.01-6.41)	2.42 (1.83-3.21)	2.41 (1.82-3.19)
**Radiotherapy target**			
Breast + axillary	1.0 (reference)	1.0 (reference)	1.0 (reference)
Breast only	0.67 (0.52-0.86)	0.91 (0.70-1.17)	0.91 (0.71-1.18)
None	1.94 (1.69-2.23)	1.55 (1.31-1.84)	1.53 (1.29-1.81)
**Diagnostic period**			
2007-2011	1.0 (reference)	1.0 (reference)	1.0 (reference)
2012-2015	0.98 (0.84-1.14)	1.17 (0.98-1.40)	1.15 (0.97-1.37)
2016-2019	0.94 (0.78-1.14)	1.12 (0.89-1.42)	1.11 (0.88-1.40)
2020-2023	1.05 (0.73-1.50)	0.96 (0.66-1.41)	1.00 (0.68-1.46)

∗ In the multivariable analysis, 70 patients (2.0% of 3495 pts) were excluded due to missing covariates (or complete case analysis was performed), *with* adjustment for diagnostic period.

*Abbreviations*: HRs, hazard ratios; PH, proportional hazard; T, tumor; N, node.

### 3.3 Prognosis of the neoadjuvant cohort

Although crude survival rates were worse for patients treated at the neoadjuvant setting (Fig. 2), there was no difference in terms of OS between patients that had received systemic therapy postoperatively versus preoperatively following exploratory propensity score matching (p = 0.39). Sensitivity analysis of patients receiving optimal treatment confirmed these results (adjusted HR = 1.04, 95% CI 0.63 – 1.73; Supplementary Fig. 3. Expectedly, patients that achieved pCR (and would thus be eligible for adjuvant pertuzumab according to ESMO and NCCN guidelines) had better outcomes than patients with residual invasive disease following neoadjuvant treatment, with corresponding 10-year OS rates of 92.3% (95% CI 81.1 – 100%) versus 78.1% (95% CI 73.0 – 83.5%), respectively (log-rank p < 0.001; Table 2).

## 4 Discussion

By analyzing a nationwide, register-based cohort of patients with HER2-positive, lymph node–positive breast cancer at initial diagnosis, we found that real-world OS was lower than the survival reported for the standard-treatment arm of the APHINITY trial (89.2% OS after a comparable 8.4 years of median follow-up for node positive patients) [14], even among patients who received optimal adjuvant therapy with chemotherapy and trastuzumab. Assuming similar proportional risk reduction with adjuvant pertuzumab as in APHINITY, the expected real-world absolute benefit should be higher due to the increased absolute risk of death. Larger tumors and more advanced nodal status identified subgroups with worse survival, underscoring the continued relevance of traditional clinicopathological factors when selecting patients for escalation strategies such as adjuvant pertuzumab.

Although comparisons across treatment cohorts are exploratory and limited by baseline differences in age and disease characteristics, several insights emerge. Patients treated with a modern neoadjuvant approach who achieved a pCR demonstrated excellent long-term outcomes compared with those in the adjuvant cohort. These findings suggest that additional post-neoadjuvant treatment intensification is unlikely to provide meaningful benefit in patients achieving pCR, even in the context of initially node-positive disease, which questions current recommendations by ESMO and NCCN for this population. This interpretation aligns with a recent real-world evidence study evaluating pertuzumab plus trastuzumab versus trastuzumab alone in patients achieving pCR, which similarly found no meaningful difference in recurrence-free survival between the two groups [15]. Moreover, in the exploratory comparison of the adjuvant cohort with patients treated with neoadjuvant therapy who did not achieve a pCR, long-term outcomes were suboptimal. A key advantage of the neoadjuvant approach is the ability to identify patients with residual disease who may benefit from post-neoadjuvant treatment escalation. This is particularly relevant in the current era, where trastuzumab deruxtecan has demonstrated substantial survival benefit in HER2-positive residual disease, further shifting the therapeutic paradigm toward response-adapted strategies. [16]. Conversely, the excellent outcomes observed in pCR patients underscore that identifying those who can safely avoid such intensification is equally critical, both to prevent unnecessary toxicity and to preserve quality of life. Our findings highlight the persistence of substantial residual risk in this population and underscore the need for improved strategies to improve patient outcomes.

The impact of nodal status and tumor size on survival outcomes was clear and consistent in our study, and highlighted a candidate population of patients with small node positive tumors for possible de-escalation of treatment by foregoing adjuvant pertuzumab since the expected absolute benefit would be marginal. On the other hand, the potential roles of ER status and tumor grade were more challenging to interpret. Although ER-positive disease was associated with superior survival overall, the relative risk of late events increased over time compared with ER-negative disease. This observation aligns with previous reports showing that ER-positive/HER2-positive breast cancer carries an increased risk of late recurrence, particularly in the presence of extensive lymph node involvement despite trastuzumab-based therapy [17,18]. Additionally, high tumor grade was linked to increased early mortality, consistent with evidence from contemporary patient cohorts treated with modern systemic therapies, in which tumor grade continues to retain its prognostic value across all breast cancer subtypes [19].

This study has several strengths, including its population-based, nationwide design and a follow-up duration sufficient to enable meaningful long-term survival analyses in patients with HER2-positive disease. The study setting, a country with an organized mammography screening program which is available to all persons registered in Sweden, as well as access to healthcare services for all individuals regardless of employment status or any other socioeconomic or demographic factors, strengthens the robustness of our results. Finally, the study period captured all significant advances in the management of HER2-positive disease, including the transition to preoperative strategies, introduction of dual HER2 blockade, and implementation of postneoadjuvant antibody-drug conjugates.

Several limitations should also be acknowledged. The relatively small number of patients in some subgroups limited statistical power and the robustness of certain analyses. Missing data for selected clinicopathological variables, together with the observational study design, may have resulted in residual confounding, particularly in comparisons that could not be fully adjusted for all relevant prognostic factors. In addition, comparisons between neoadjuvant and adjuvant treatment settings are potentially susceptible to immortal-time bias, which may favor the neoadjuvant cohort. To mitigate this risk, time zero was uniformly defined as the date of surgery in all survival analyses. The effectiveness of pertuzumab could not be directly evaluated because only a small proportion of patients received this therapy. Consequently, our findings should be interpreted as indirect evidence reflecting differences in baseline mortality risk rather than as definitive estimates of treatment effectiveness. Furthermore, the study period was prolonged which could introduce bias not fully accounted for with adjustment for calendar period, caused by temporal differences in diagnostic strategies, indication for preoperative therapy and introduction of novel treatments to clinical routine. Finally, trastuzumab usage was relatively low in our study and more so in the initial study period. While some degree of missing treatment data cannot be excluded, our findings may also reflect the expected similar non-total usage of pertuzumab, which might limit its impact on outcomes at the population level.

In summary, this nationwide real-world cohort demonstrates that patients with HER2-positive, node-positive breast cancer continue to experience poorer survival than those enrolled in clinical trials, even after exposure to trastuzumab-based adjuvant therapy. Tumor size and nodal burden remain the most influential prognostic factors and can guide decisions regarding treatment escalation. Adjuvant pertuzumab could potentially provide meaningful benefit mainly to a small subset of patients with high baseline risk who do not receive optimal neoadjuvant therapy, supporting a risk-adapted approach in clinical practice. Although fewer HER2-positive, node-positive patients now undergo primary surgery, suboptimal long-term outcomes at the population level highlight the persistent unmet need for effective escalation strategies in selected high-risk patients. Conversely, our hypothesis-generating results indicate that patients achieving a pCR after neoadjuvant therapy have excellent long-term prognosis and may be currently overtreated, which motivates further studies specifically designed to answer the question of treatment de-escalation.

## Data sharing statement

The dataset supporting the findings of this study is available from the National Quality Registry for Breast Cancer (NKBC) upon request (https://cancercentrum.se/diagnosbehandling/cancerdiagnoser/brost/kvalitetsregister.7359.html).

## Funding

This study received no specific funding.

## CRediT authorship contribution statement

**Antonis Valachis:** Conceptualization, Supervision, Writing – original draft, Writing – review & editing. **Xingrong Liu:** Data curation, Formal analysis, Visualization, Writing – original draft, Writing – review & editing. **Balazs Acs:** Project administration, Resources, Writing – review & editing. **Johan Hartman:** Project administration, Resources, Writing – review & editing. **Theodoros Foukakis:** Project administration, Resources, Writing – review & editing. **Alexios Matikas:** Conceptualization, Methodology, Supervision, Writing – original draft, Writing – review & editing.

## Declaration of competing interest

The authors declare that they have no known competing financial interests or personal relationships that could have appeared to influence the work reported in this paper.
