# Supplementary material for: A nationwide population-based study on the incidence and prognosis of HER2-positive breast cancer eligible for adjuvant pertuzumab in the modern era of neoadjuvant therapy
Source: Breast. 2026 Jun 8;88:104834. doi: 10.1016/j.breast.2026.104834 (PMC13277662; doi:10.1016/j.breast.2026.104834)
Supplement: Multimedia component 1 [file mmc1.docx]

| **Supplementary Table 1.** Distribution of baseline characteristics and treatments for **2119** patients who had been treated with trastuzumab in combination with chemotherapy after upfront surgery. Percentages are calculated on complete data. | |
| --- | --- |
|  | **Subset of the adjuvant cohort**  n (%) |
| **No. of patients** | 2119 |
| **Diagnostic age, yrs,** median (Q1-Q3) | 58 (48-67) |
| < 40 | 170 (8.0) |
| 40-49 | 423 (20.0) |
| 50-64 | 860 (40.6) |
| 65-79 | 604 (28.5) |
| ≥ 80 | 62 (2.9) |
|  |  |
| **Estrogen receptor** |  |
| Negative | 714 (33.8) |
| Positive | 1398 (66.2) |
| Missing | 7 |
|  |  |
| **Pathological tumor size** |  |
| T1 | 926 (44.0) |
| T2 | 1025 (48.7) |
| T3 | 152 (7.2) |
| Missing | 16 |
|  |  |
| **Pathological nodal status** |  |
| N1 | 1471 (69.4) |
| N2 | 443 (20.9) |
| N3 | 205 (9.7) |
|  |  |
| **Pathological tumor grade **** |  |
| Grade 1-2 | 648 (30.6) |
| Grade 3 | 1471 (69.4) |
|  |  |
| **Ki-67 expression, %** |  |
| Median (Q1-Q3) | 40.0 (27.0-53.0) |
| Missing | 609 |
|  |  |
| **Type of adjuvant chemotherapy** |  |
| Taxane- and anthracycline-based | 908 (42.9) |
| Taxane only | 185 (8.7) |
| Anthracycline only | 894 (42.2) |
| None (or missing) | 132 (6.2) |
|  |  |
| **Adjuvant pertuzumab** (administrated) | 77 (3.6) |
|  |  |
| **Adjuvant radiotherapy target** |  |
| Breast & Axillary | 1500 (70.8) |
| Breast only | 364 (17.2) |
| None (or missing) | 255 (12.0) |
|  |  |
| **Endocrine therapy** |  |
| Administrated | 1400 (66.1) |
| Missing | 1 |

| **Supplementary Table 2.** Test of proportional hazard assumptions using a multivariable Cox model, based on **2119** patients who had been treated with trastuzumab in combination with chemotherapy after upfront surgery | | | |
| --- | --- | --- | --- |
| Multivariable analysis **with** adjustment for diagnostic period | | | |
|  | **Chi-square value** | **Degree of freedom** | **P-value** |
| Age | 1.115 | 1 | 0.2911 |
| **ER-status** | **18.951** | **1** | **1.3e-05** |
| **Grade** | **7.097** | **1** | **0.0077** |
| Tumor size | 2.892 | 2 | 0.2355 |
| Nodal status | 0.984 | 2 | 0.6113 |
| Chemotherapy type | 6.541 | 3 | 0.0881 |
| Radiotherapy | 0.679 | 2 | 0.7120 |
| Diagnostic period | 5.256 | 3 | 0.1540 |
| *GLOBAL* | 39.820 | 15 | 0.0005 |
| Multivariable analysis **without** adjustment for diagnostic period | | | |
|  | **Chi-square value** | **Degree of freedom** | **P-value** |
| Age | 1.248 | 1 | 0.2640 |
| **ER-status** | **18.830** | **1** | **1.4e-05** |
| **Grade** | **7.003** | **1** | **0.0081** |
| Tumor size | 2.803 | 2 | 0.2463 |
| Nodal status | 1.0111 | 2 | 0.6031 |
| Chemotherapy type | 6.772 | 3 | 0.0795 |
| Radiotherapy | 0.635 | 2 | 0.7278 |
| *GLOBAL* | 33.797 | 12 | 0.0007 |

| **Supplementary Table 3.** Univariate and multivariable analysis for association of baseline characteristics with overall survival on **2119** patients who had been treated with adjuvant *trastuzumab* in combination with chemotherapy after upfront surgery | | | |
| --- | --- | --- | --- |
|  | **Hazard ratio (95% CI)** | | |
|  | Univariate analysis, PH models | Multivariable analysis, PH model 1 | Multivariable analysis, non-PH model 1 |
| **Age, yrs** | 1.05 (1.04-1.06) | 1.04 (1.03-1.05) | 1.04 (1.03-1.05) |
|  |  |  |  |
| **Estrogen receptor** |  |  |  |
| Negative | 1.0 (reference) | 1.0 (reference) | 1.0 (reference) |
| Positive | 0.77 (0.62-0.96) | 0.96 (0.77-1.21) | Time-dependent HRs  *at 2 years since surgery:*  0.61 (0.44-0.86) *at 5 years since surgery:*  1.08 (0.83-1.40)  *at 8 years since surgery:*  1.61 (1.13-2.30) *at 10 years since surgery:*  1.95 (1.20-3.17) |
|  |  |  |  |
| **Tumor grade** |  |  |  |
| Grade 1-2 | 1.0 (reference) | 1.0 (reference) | 1.0 (reference) |
| Grade 3 | 1.68 (1.29-2.17) | 1.48 (1.13-1.95) | Time-dependent HRs  *at 2 years since surgery:*  2.08 (1.33-3.25) *at 5 years since surgery:*  1.67 (1.25-2.23)  *at 8 years since surgery:*  1.23 (0.88-1.71) *at 10 years since surgery:*  1.05 (0.70-1.58) |
|  |  |  |  |
| **Tumor size** |  |  |  |
| T1 | 1.0 (reference) | 1.0 (reference) | 1.0 (reference) |
| T2 | 1.79 (1.40-2.29) | 1.34 (1.04-1.72) | 1.35 (1.05-1.73) |
| T3 | 3.48 (2.48-4.88) | 2.53 (1.78-3.59) | 2.50 (1.76-3.55) |
|  |  |  |  |
| **Nodal status** |  |  |  |
| N1 | 1.0 (reference) | 1.0 (reference) | 1.0 (reference) |
| N2 | 1.91 (1.50-2.44) | 1.73 (1.33-2.25) | 1.75 (1.35-2.28) |
| N3 | 3.13 (2.37-4.13) | 2.50 (1.86-3.36) | 2.47 (1.84-3.33) |
|  |  |  |  |
| **Chemotherapy type** |  |  |  |
| Anthracycline and Taxane | 1.0 (reference) | 1.0 (reference) | 1.0 (reference) |
| Anthracycline only | 1.53 (1.16-2.03) | 1.32 (0.996-1.76) | 1.28 (0.97-1.71) |
| Taxane only | 3.51 (2.40-5.14) | 1.86 (1.24-2.79) | 1.79 (1.19-2.70) |
| None | 2.95 (2.02-4.30) | 1.88 (1.28-2.77) | 1.84 (1.25-2.71) |
|  |  |  |  |
| **Radiotherapy target** |  |  |  |
| Breast + axillary | 1.0 (reference) | 1.0 (reference) | 1.0 (reference) |
| Breast only | 0.59 (0.41-0.84) | 0.83 (0.57-1.19) | 0.83 (0.57-1.20) |
| None | 1.66 (1.27-2.17) | 1.94 (1.45-2.58) | 1.93 (1.45-2.57) |
|  |  |  |  |
| **Diagnostic period** |  |  |  |
| 2007-2011 | 1.0 (reference) | 1.0 (reference) | 1.0 (reference) |
| 2012-2015 | 1.05 (0.82-1.35) | 1.09 (0.83-1.42) | 1.06 (0.82-1.38) |
| 2016-2019 | 0.79 (0.56-1.10) | 0.74 (0.47-1.16) | 0.73 (0.47-1.14) |
| 2020-2023 | 0.92 (0.45-1.85) | 1.05 (0.48-2.27) | 1.17 (0.54-2.54) |
| * In the multivariable analysis, 43 patients (2.0% of 2119 pts) were excluded due to missing covariates (or complete case analysis was performed), *without* adjustment for diagnostic period.  *Abbreviations*: HRs, hazard ratios; PH, proportional hazard; T, tumor; N, node. | | | |

**Supplementary Figure 1.** Number of patients with HER2-positive **(A)** and HER2-positive and node positive **(B)** breast cancer. **(C)** and **(D)** present respective number of patients in the neoadjuvant setting. The annual proportion of patients with primary resected, node positive, HER2-posirive (APHINITY eligibility) in relation to the total HER2-positive population is shown in **(E)**.


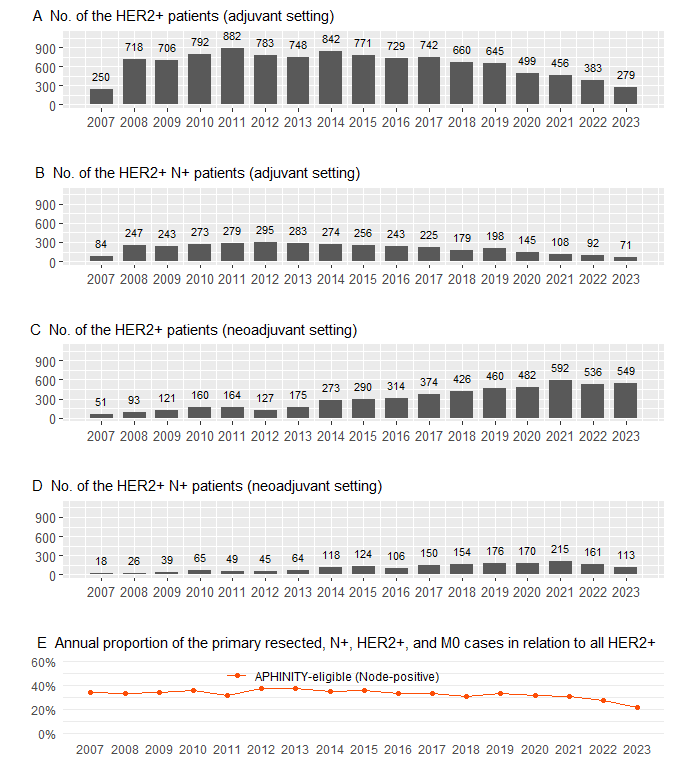


**Supplementary Figure 2.** **(A)** Kaplan-Meier survival curves with log-rank test comparing overall survival of patients treated with adjuvant treatment, in age-defined subgroups; and **(B)** Same analysis confined to patients that received adjuvant chemotherapy and trastuzumab.


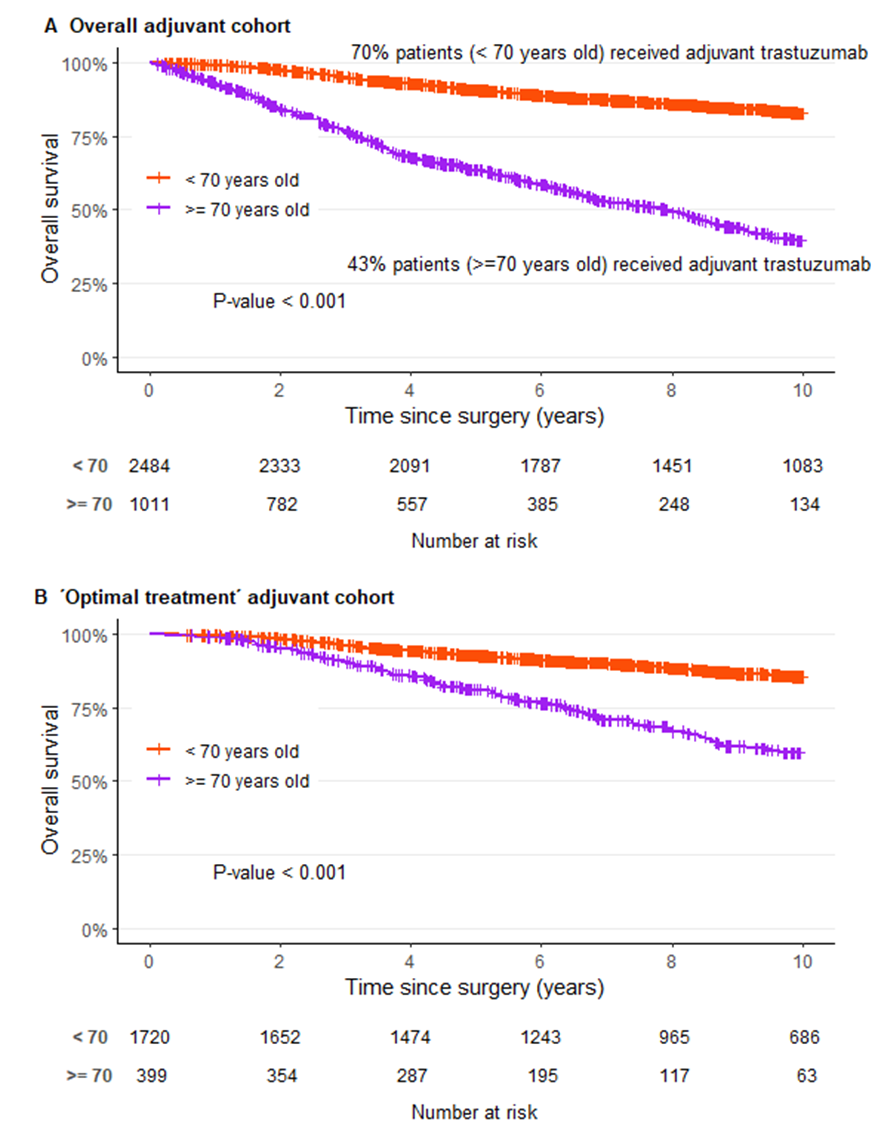


**Supplementary Figure 3.** **(A)** Kaplan-Meier survival curves with log-rank test comparing overall survival of patients treated with adjuvant versus neoadjuvant treatment; and **(B)** multivariable-adjusted survival curves (adjusted for age, TNM stages, and histologic grades from surgical specimen), reported with adjusted hazard ratio (95% CI).


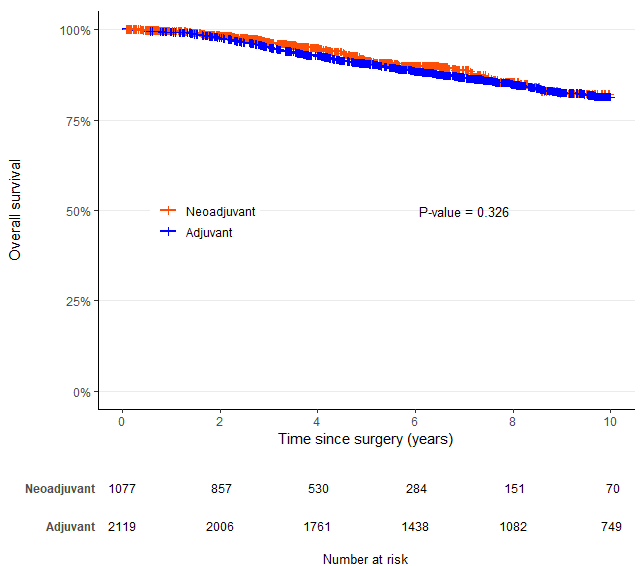
A


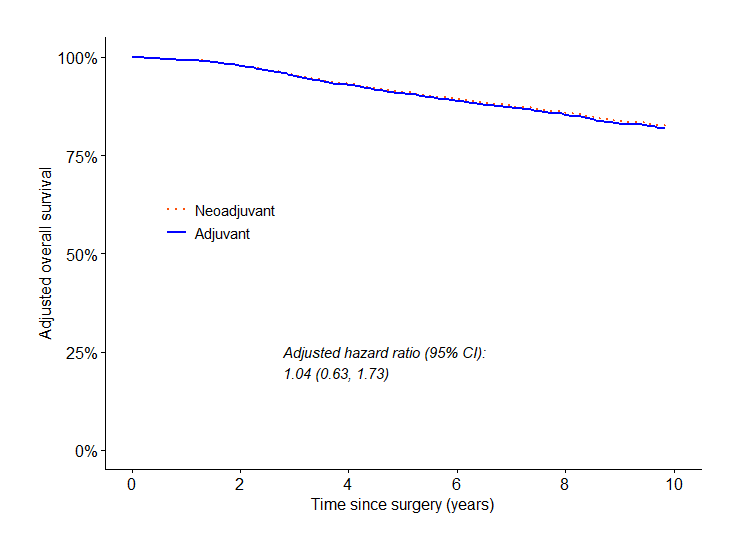


B
